# Emerging Small Science on Nanomaterials for Energy Storage and Catalysis

**DOI:** 10.1002/smsc.202100101

**Published:** 2021-10-20

**Authors:** Shaojun Guo, Qiang Zhang, Shuangyin Wang

**Affiliations:** ^1^ School of Materials Science & Engineering Peking University Beijing 100871 China; ^2^ Beijing Key Laboratory of Green Chemical Reaction Engineering and Technology Department of Chemical Engineering Tsinghua University Beijing 100084 China; ^3^ State Key Laboratory of Chem/Bio-Sensing and Chemometrics College of Chemistry and Chemical Engineering Hunan University Changsha P. R. China

1

Renewable energy plays the key role in achieving green economy by addressing global energy crisis. Energy conversion and storage technologies can implement the generation and utilization of renewable energy based on the photochemical or electrochemical processes. In these sustainable processes, energy can physically/chemically release/store in various matters (electrode, fuel, etc.), resulting in the rapid emergence of nanomaterials with adjustable performance for energy storage and catalysis. In particular, rechargeable batteries and electro(photo)‐catalysis are of significance for achieving efficient utilization of energy, high‐performance fuel cells, water electrolyzers and high value‐added compounds synthesized from CO_2_ or available feedstock by rationally designing nanoscale electrode materials and high‐performance catalysts.

This special issue of *Small Science* affords a series of high‐quality research and review articles in the fields of nanomaterials for energy storage and catalysis. It will be published as issue 10 and issue 11, including 5 research articles and 8 reviews, which cover very recent progress in alkali metal batteries, photocatalysis, electrocatalysis, and electrosynthesis. These topics collected in this special issue can afford valuable references for developing and designing advanced functional nanomaterials for efficient and effective energy storage and conversion.

Rechargeable batteries with alkali metal anodes are promising alternatives to routine lithium‐ion batteries. The solid electrolyte interphase (SEI) is an important research topic for Li‐based secondary batteries. Starting with the fundamental knowledge on the structure of SEI, Anmin Cao and co‐workers discussed emerging strategies for constructing the fluorides‐based interfaces, including the surface coating of metal fluorides on cathodes and ex situ/in situ fluorination on lithium. To observe the SEI layer at the nanoscale or even atomic scale, the cryo‐EM provides new opportunities in terms of maintaining the native states. In this case, the key contribution of cryo‐EM to characterize sensitive battery materials were summarized by Xinyong Tao and co‐workers. Jia‐Qi Huang and co‐workers also comprehensively review recent advances on Li metal batteries, including the possible thermal runaway mechanism, the measurement methods of thermal stability and nonflammability of liquid electrolytes.

Compared with traditional lithium‐ion batteries, alkali‐metal/sulfur batteries hold great potential for achieving high energy density. Although electrochemical impedance spectroscopy (EIS) analysis can reasonably provide detailed information for the conversion reactions of lithium polysulfides (LiPSs), a reasonable explanation on the EIS responses is still ambiguous due to the lack of a well‐defined equivalent circuit. Herein, Qiang Zhang and co‐workers systematacially analyzed the EIS responses of LiPS symmetric cells, and propose a well‐defined equivalent circuit to accurately interpret key parameters. In addition to lithium–sulfur batteries, the emerging sodium‐sulfur batteries have also become a new family of alkali‐metal/S batteries. Yan Yu and co‐workers review the rationally designed strategies of combining porous carbon with “adsorption‐catalysis” agents, including transition‐metal single‐atom, transition‐metal nanoclusters, transition‐metal compounds, and heterostructures. The multi‐step reaction mechanism of the electrochemical reaction of various sodium polysulfides during the redox process are also summarized.

Carbon dioxide (CO_2_), as a typical greenhouse gas, has become the culprit of global warming. The conversion of CO_2_ into fuels is an emerging solution to address this environmental issue. The visible‐light driven CO_2_ reduction reaction (CO_2_RR) provided a feasible strategy to reduce CO_2_ into chemical fuels *via* the photocatalytic reaction. Liqiang Jing and co‐workers reported a class of ultrathin copper phthalocyanine (CuPc)/α‐Fe_2_O_3_ heterojunction for reducing CO_2_ to CO and CH_4_ with high photoactivity. In addition, electrochemical reduction of CO_2_ into value‐added chemical fuels is another important technology towards carbon‐neutral economy. To this end, a novel strategy for synthesizing highly dispersed indium oxide nanoparticles supported on carbon nanorods was presented by Yanguang Li and co‐workers. The well‐designed electrocatalysts showed high reactivity for reducing CO_2_ into formate with high Faradaic efficiency. Gengfeng Zheng and co‐workers overview recent advances in electrochemical CO_2_RR catalysts and the corresponding reaction mechanisms. These works are highly expected to provide insightful understanding for facilitating the development of CO_2_RR reaction mechanisms and optimizing high reactivity and selectivity electrocatalysts towards CO_2_RR.

The production and utilization of hydrogen (H_2_) is another important strategy to achieve sustainable economy. This is highly dependent on two key technologies, electrochemically overall water splitting and fuel cells. To obtain high‐performance acidic water oxidation electrocatalysis, Sen Zhang and co‐workers designed three kinds of molecular cyclopentadienyl iridium(III) complexes as precursors for electrochemical oxygen evolution reaction (OER) catalysts. Gang Wu and co‐workers described a new electrocatalyst with unique core/shell structures, a polymerized FeTPPCl‐derived carbon layer (*p*‐FeNC) on the Co‐doped ZIF‐derived carbon (CoNC), which exhibited excellent activity and stability towards oxygen reduction reaction (ORR) and CO_2_RR. As a typical nonplatinum (non‐Pt) catalyst, palladium (Pd)‐based catalysts attract increasing attentions in various electrocatalytic reactions. Using the typical electrocatalytic reactions as probes, Shaojun Guo and co‐workers review recent progress in advanced structural regulation strategies for enhancing electrocatalytic performance. Integrating two key electrode reactions, OER and ORR, rechargeable zinc‐air batteries can be assembled to exhibit potential application for next‐generation energy conversion and storage technologies. Yang Yang and co‐workers discuss recent advances on the metal anode and air cathode of zinc‐air batteries.

The electrosynthesis of high‐value‐added products has attracted increasing attention to replace the traditional industrial production of chemicals in the field of energy conversion. Especially electrochemical urea synthesis is a very important reaction. Shuangyin Wang and co‐workers review recent progress on green urea synthesis by concentrating on the electrocatalytic coupling of carbon source (carbon dioxide) and nitrogen source (nitrogen, nitrite, nitrate) for direct urea synthesis under mild ambient conditions.

This special issue in *Small Science* will afford an international platform for promoting the experts’ cooperation between materials science, chemistry, physics and environmental sciences. It is expected that advanced nanomaterials will provide a strong platform for promoting the development of renewable energy. Finally, we would like to express our sincere thanks to the editorial team of *Small Science*, especially Dr. Lu Shi, for providing the precious opportunity to publish this special issue and their efforts for handling all the manuscripts. We also would like to thank all the authors and reviewers for their excellent contributions to these high‐quality works.

